# Identification of 2,3-oxidosqualene cyclase gene in *Eleutherococcus senticosus* and its regulatory mechanism in saponin synthesis

**DOI:** 10.1093/hr/uhaf133

**Published:** 2025-05-21

**Authors:** Yaqi Cui, Jiacheng Ma, Mengying Jiao, Xueying Zhao, Jingwen Ding, Chenran Feng, Peng Liu, Yuehong Long, Zhaobin Xing

**Affiliations:** College of Life Sciences, North China University of Science and Technology, Tangshan 063210, China; College of Life Sciences, North China University of Science and Technology, Tangshan 063210, China; College of Life Sciences, North China University of Science and Technology, Tangshan 063210, China; College of Life Sciences, North China University of Science and Technology, Tangshan 063210, China; College of Life Sciences, North China University of Science and Technology, Tangshan 063210, China; College of Life Sciences, North China University of Science and Technology, Tangshan 063210, China; College of Life Sciences, North China University of Science and Technology, Tangshan 063210, China; College of Life Sciences, North China University of Science and Technology, Tangshan 063210, China; College of Life Sciences, North China University of Science and Technology, Tangshan 063210, China

## Abstract

Oleanane-type triterpenoid saponins are the primary medicinal components of *Eleutherococcus senticosus*. During saponin biosynthesis in *E. senticosus*, various members of the 2,3-oxidosqualene cyclase (OSC) gene family can direct 2,3-oxidosqualene into triterpene saponin and sterol synthesis pathways. However, the precise molecular mechanism underlying this phenomenon remains unclear. We initially screened for β-amyrin synthase 1 (bAS1) and cycloartenol synthase 1 (CAS1) among 10 *EsOSC* genes using genome-wide identification and correlation analysis. Subcellular localization, catalytic experiments, and *in vivo* transient overexpression demonstrated that *Es*bAS1 and *Es*CAS1 catalyze the formation of the triterpene skeleton β-amyrin and sterol precursor cycloartenol exclusively in the cytoplasm, enhancing and inhibiting the *in vivo* biosynthesis of oleanane-type saponins, respectively. Results from site-directed mutagenesis and molecular docking indicated that W-WY and Y-WH triplets characterized the active sites of *EsbAS1 and EsCAS1*, respectively. GUS (β-glucuronidase) staining and electrophoretic mobility shift assay (EMSA) experiments on the promoter region revealed that various colored light quality, DNA methylation, and five transcription factors (*Es*NAC047, *Es*NAC098, *Es*WRKY40, *Es*MYB4, and *Es*ERF66) regulated the expression of *EsbAS1* and *EsCAS1*. This study provides preliminary insights into the molecular mechanisms by which *EsbAS1* and *EsCAS1* regulate saponin synthesis in *E. senticosus*.

## Introduction

*Eleutherococcus senticosus* (Rupr. & Maxim.) Maxim is a traditionally valued Chinese medicinal plant that belongs to the Araliaceae family, which also includes *Panax ginseng* [1]. *Eleutherococcus senticosus* contains a variety of chemical constituents, among which triterpenoid saponins are one of its main active components [2]. These triterpenoids serve dual purposes: they protect plants from pathogens and enhance their defenses [3]. In addition, they exhibit a wide range of pharmacological activities that are beneficial to humans [4].

Genome sequencing results have indicated that the genome of *E. senticosus* contains 36 372 coding genes [5]. Transcriptome sequencing results, combined with metabolomic analysis, have demonstrated that drought stress [6], light stimulation [,7], and growth and developmental status [8, 9] influence the expression of genes associated with triterpene saponin synthesis and the accumulation of metabolites in *E. senticosus*. By synthesizing the findings from omics analysis [6–8], the following biosynthetic pathway of triterpenoid saponins in *E. senticosus* was identified:

Triterpenoid components are synthesized in *E. senticosus,* primarily through the mevalonate (MVA) pathway. In this pathway, squalene is oxidized by squalene epoxidase (SE) to form 2,3-oxidosqualene [5, 9, 10]. This compound is then catalytically converted by 2,3-oxidosqualene cyclase (OSC) into a variety of triterpenoid saponin skeletons with distinct conformations and structures, which serve as the foundation for functionally diverse triterpenoid components [11]. According to the classification based on the OSC function, the majority of the identified enzymes are β-amyrin synthases (bAS), which are responsible for synthesizing the triterpene saponin precursor β-amyrin. Additionally, some enzymes are classified as cycloartenol synthases (CAS), which synthesize the phytosterol precursor cycloartenol. The OSC enzymes possess a highly conserved catalytic center, where specific key amino acid residues can influence the products generated by OSC. For instance, a mutation in a crucial residue (N612K) of bAS (CqbAS1) in *Chenopodium quinoa* leads to the production of B,C-ring-opening triterpenes [12]. These investigations primarily utilize synthetic biology approaches that modify the triterpene skeleton structure and consequently alter the triterpenoids by targeting key amino acid residues within OSC. However, there is a scarcity of research focused on regulating OSC gene expression at the gene expression level.

All biosynthetic genes involved in the synthesis of triterpenoid saponins via the MVA pathway in *E. senticosus* have been cloned, with *Es*FPS, *Es*SS, and *Es*SE identified as key enzymes [6, 7, 13]. Further research has indicated that the synthesis of triterpenoid saponins in *E. senticosus* correlates with DNA methylation in response to drought stress [6, 9, 14, 15]. Bisulfite sequencing has revealed that cytosine DNA methylation in the *Es*FPS, *Es*SS, and *Es*SE promoters [9] as well as in the mevalonate diphosphate decarboxylase (*MDD*) gene, significantly affects gene expression and saponin synthesis [8]. However, even among *E. senticosus* specimens with similar saponin content, there were significant differences in DNA methylation ratios [6, 9]. This suggests that functional genes other than those mentioned above may play crucial roles in the synthesis of triterpenoid saponins in *E. senticosus*.

According to the biosynthetic pathway of triterpenoid saponins, 2,3-oxidosqualene, catalyzed by the cascade of *Es*FPS, *Es*SS, and *Es*SE, must undergo a cyclization reaction catalyzed by *Es*OSC to form a cyclized carbon chain structure, which subsequently leads to the formation of various triterpenoid saponins [7, 12]. Both bAS and CAS from *E. senticosus* are members of the *EsOSC* gene family. *Es*bAS can enter the triterpene saponin metabolic pathway by cyclizing 2,3-oxidosqualene to create a triterpene saponin carbon skeleton after β-amyrin formation [16]. In contrast, *Es*CAS catalyzes the cyclization of 2,3-oxidosqualene to cycloartenol, which then enters the sterol synthesis pathway [17]. For example, *Ws*CAS from *Withania somnifera* exclusively directs 2,3-oxidosqualene toward the sterol synthesis pathway. Overexpression or silencing of *WsCAS* leads to an increase or decrease in sterol levels [18]. Elevated expression of *Pg*bAS in *P. ginseng* reduces sterol synthesis and facilitates the entry of more 2,3-oxidosqualene into the triterpene saponin synthesis pathway [17]. This suggests that *Es*bAS and *Es*CAS, members of the *EsOSC* gene family, compete for substrate 2,3-oxidosqualene and serve as key rate-limiting enzymes in the synthesis of triterpenoid saponins in *E. senticosus*.

2,3-oxidosqualene cyclase belongs to a superfamily of enzymes that are highly conserved in plants [19]. This enzyme catalyzes the protonation, cyclization, rearrangement, and deprotonation of 2,3-oxidosqualene, leading to the formation of triterpene skeletons, such as β-amyrin, lupeol, and friedelin. Additionally, after the formation of cycloartenol, it ultimately contributes to the synthesis of sterols [12, 18]. Therefore, identification and functional analysis of the *EsOSC* gene family are crucial for elucidating the molecular mechanisms underlying the differences in saponin content in *E. senticosus*.

In this study, we comprehensively identified the *EsOSC* gene family in *E. senticosus* and confirmed the catalytic enzymes *Es*bAS and *Es*CAS using bioinformatic and molecular biology methods. In addition, we preliminarily elucidated the conserved motifs of their catalytic activities and regulators of gene expression. These findings establish a foundation for further investigation of the molecular mechanisms by which *EsOSCs* regulate saponin synthesis in *E. senticosus*.

## Results

### Genome-wide identification and structural analysis of *EsOSCs*

**Figure 1:**
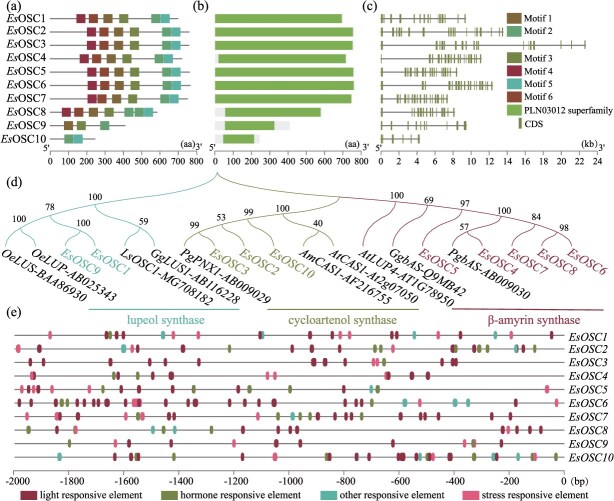
**Genome-wide identification of *EsOSCs*.** (**a**) Conserved motifs of *Es*OSC. (**b**) Conserved structural domains of *Es*OSC. (**c**) Gene structure of *EsOSC.* (**d**) Phylogenetic analysis of *Es*OSC. (**e**) Cis-acting elements of the *EsOSC* promoter.

Ten *EsOSC* genes, distributed across eight chromosomes, were identified in the genome of *E. senticosus* (Fig. S1) and designated as *EsOSC1–10* (Table S3). The *EsOSC* gene family contains six conserved motifs (Motifs 1–6). *Es*OSC1–8 contained all motifs, *Es*OSC9 included only Motifs 1–3, and *Es*OSC10 contained only motifs 2 and 5 (Fig. 1a and Fig. S2). The conserved structural domains of *Es*OSC were notably specific, showing that the full-length protein sequences are all conserved structural domain of the PLN03012 superfamily (Fig. 1b). All *EsOSC* genes lack a UTR region and consist solely of CDS and intron sequences. *EsOSC4–8* exhibited similar structural characteristics; the CDS region was concentrated in the posterior half, suggesting that these four structurally analogous genes may have evolved from a common ancestor (Fig. 1c and d). Phylogenetic analysis (Fig. S3) revealed that *EsOSCs* can be classified into three subfamilies: five belonging to the bAS synthase subfamily, three to the CAS synthase subfamily, and two to the lupeol synthase subfamily (Fig. 1d, Table S4). The covariance of *EsOSCs* was more pronounced within the same subfamily (Fig. S4). The *cis*-acting elements of the *EsOSC* promoter were primarily categorized as light-responsive, hormone-responsive, stress-responsive, and other response elements, with 134 being identified as light-responsive elements (Fig. 1e, Table S5).

### Screening for primary *EsbAS* and *EsCAS*

Blast comparison of the gene sequences of these 10 *OSC* with the transcriptome data revealed that six *OSC* genes corresponded to the same transcriptome sequence, resulting in a total of four distinct transcriptome sequences (Cluster-90580.10, Cluster-95661.5, Cluster-99183.11, Cluster-90580.9). Consequently, it is concluded that six of the 10 *OSC* genes are highly duplicated, leaving only four normally transcribed *OSC* genes.

**Figure 2:**
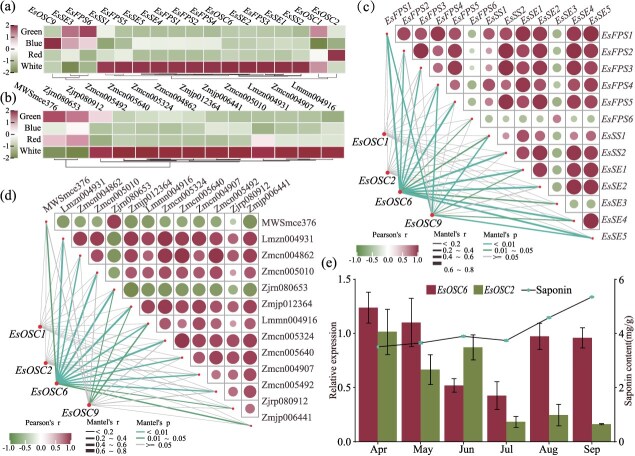
**Screening of *EsbAS* and *EsCAS*.** (**a**) Expression of saponin synthase genes in *E. senticosus* under different light conditions. (**b**) Triterpenoid metabolite content in *E. senticosus* under different light conditions. (**c**) Correlation of *EsOSC* and the expression of genes involved in saponin biosynthesis enzymes. (**d**) Correlation of *EsOSC* with the content of saponins. (**e**) Expression of *EsOSC2* and *EsOSC6* genes and saponin content in *E. senticosus* at different growth periods.

Transcriptome sequencing analysis revealed that only *EsOSC1*, *EsOSC2*, *EsOSC6*, and *EsOSC9*, out of a total of 10 *EsOSCs*, exhibited normal transcription levels in *E. senticosus* (Fig. 2a). Consequently, these four *EsOSCs* are likely to be involved in the biosynthesis of saponins in *E. senticosus*. To further elucidate the specific functions of these four *EsOSCs*, we separately assessed the contents of triterpenoid saponin metabolites (Fig. 2b, Table S6) and the expression levels of their biosynthesis-related genes (Fig. 2a) in the corresponding samples. Correlation analysis indicated a relationship between all four *EsOSCs* and the expression of saponin biosynthetic enzyme genes in *E. senticosus*. Notably, the expression of *EsOSC2* was significantly negatively correlated (*P* < 0.01) with the expression of *EsFPS*, *EsSS*, and *EsSE*, whereas that of *EsOSC6* was significantly positively correlated (*P* < 0.01) with *EsFPS*, *EsSS*, and *EsSE* (Fig. 2c). Similar to the correlation observed in gene expression levels, *EsOSC2* exhibited a significant negative correlation (*P* < 0.01) with the quantity of saponin metabolites, whereas *EsOSC6* demonstrated a significant positive correlation (*P* < 0.01) with these metabolites (Fig. 2d). Further analysis of the expression levels of *EsOSC2* and *EsOSC6*, along with the triterpenoid saponin content in *E. senticosus* during different growth stages (Fig. 2e), revealed that triterpenoid saponins gradually accumulated with the growth of *E. senticosus*, and the expression of *EsOSC6* was positively correlated with saponin accumulation (*P* < 0.05). The expression of *EsOSC2* was higher during the early stages of organ formation in *E. senticosus* but decreased significantly thereafter. Overall, these two genes exhibited a significant negative correlation (*P* < 0.01).

By integrating the results from the phylogenetic tree (Fig. 1d) and correlation analysis, it can be inferred that *EsOSC2* corresponds to *EsCAS*, which catalyzes sterol synthesis (designated as *EsCAS1*), whereas *EsOSC6* corresponds to *EsbAS*, which catalyzes saponin synthesis (designated as *EsbAS1*).

### Subcellular localization of *Es*bAS1 and *Es*CAS1

**Figure 3:**
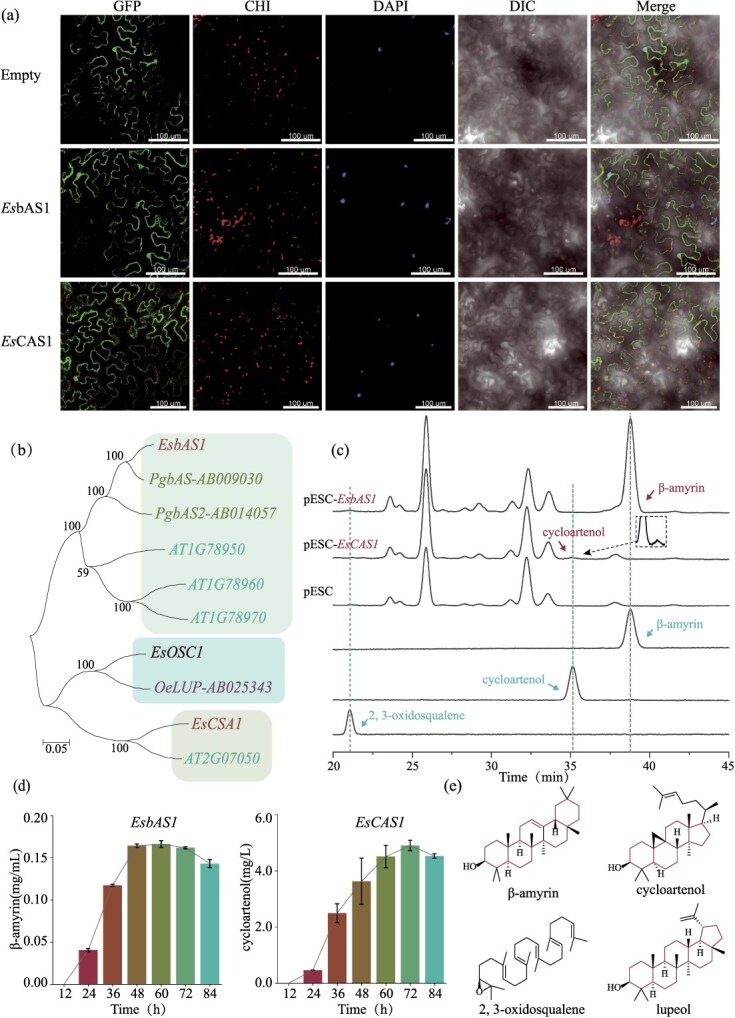
**Subcellular localization and activity analysis of *Es*bAS1 and *Es*CAS1.** (**a**) Subcellular localization of *Es*bAS1 and *Es*CAS1. (**b**) Analysis of the type to which *Es*bAS1 and *Es*CAS1 belong. (**c**) Analysis of the catalytic function of *Es*bAS1 and *Es*CAS1. (**d**) Analysis of the catalytic activity of *Es*bAS1 and *Es*CAS1. (**e**) Structure of the compounds involved.

GFP refers to the green fluorescence field, with an excitation light wavelength of 488 nm. CHI denotes the chloroplast autofluorescence field, while DAPI signifies the DAPI field, which has an excitation light wavelength of 405 nm. DIC stands for bright field, and Merge indicates the superimposed field. The transient expression of the empty vector exhibited green fluorescence in both the cytoplasm and the nucleus. Transient expression of the open reading frames (ORFs) of *EsbAS1* and *EsCAS1* fused with green fluorescent protein genes (Table S7) demonstrated green fluorescence in the cytoplasm and a portion of the nucleus within the epidermis of *Nicotiana benthamiana* (Fig. 3a). This observation indicated that *Es*bAS1 and *Es*CAS1 primarily perform their biological functions in the cytoplasm.

### Functional characterization of *Es*bAS1 and *Es*CAS1

The results of catalysis experiments conducted on αAM2 strains with high expression of the substrate 2,3-oxidosqualene indicated that *Es*bAS1, which clusters with other bAS (Fig. 3b), catalyzes the production of a single product, β-amyrin. In contrast, *Es*CAS1, which clusters with other CAS (Fig. 3b), catalyzed the production of a single product, cycloartenol (Fig. 3c). This finding suggests that *Es*bAS1 exclusively catalyzes the conversion of 2,3-oxidosqualene to β-amyrin, thereby entering the saponin metabolic pathway, whereas *Es*CAS1 exclusively catalyzes the conversion to cycloartenol, thus entering the sterol metabolic pathway.

The detection of metabolites at various incubation times is illustrated in Fig. 3d and e, and Fig. S5. *Es*bAS1-catalyzed production of β-amyrin stabilized at 48 h, reaching a maximum concentration of 0.166 mg/ml at 60 h. In contrast, *Es*CAS1-catalyzed production of cycloartenol peaked at 72 h at a concentration of 4.909 mg/l.

### Conserved amino acid analyses of *Es*bAS1 and *Es*CAS1

**Figure 4:**
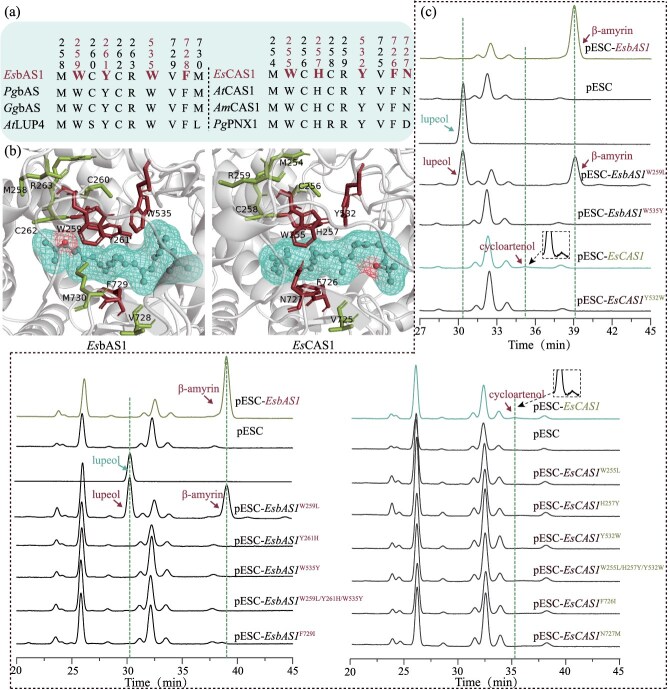
**Conserved amino acid analysis of *Es*bAS1 and *Es*CAS1.** (**a**) Conserved amino acids of OSC. (**b**) Molecular docking of *Es*bAS1 and *Es*CAS1 with 2,3-oxidosqualene. (**c**) HPLC peak profiles of the catalytic products of *Es*bAS1 with *Es*CAS1 and its mutants.

The results of the protein sequence comparison are shown in Figs S6 and S7. The bAS has highly conserved MWCYCR and VFM motifs, whereas the CAS has conserved MWCHCR and VFN motifs. Additionally, bAS and CAS possessed highly conserved amino acids, specifically W535 and Y532, respectively (Fig. 4a). Further molecular docking model and molecular dynamics simulations analyses revealed that residues M258, C260, C262, R263, V729, and M730 in the MWCYCR, VFM motif are spatially distant from the small molecule 2,3-oxidosqualene. Similarly, residues M254, C256, C258, R259, and V725 in the MWCHCR, VFN motif are spatially distant from the small molecule 2,3-oxidosqualene. But the residues W259, Y261, W535, and F729 in *Es*bAS1 and W255, H257, Y532, F726, and N727 in *EsCAS1* surround the substrate 2,3-oxidosqualene (Fig. 4b) [12, 20]. The results of molecular dynamics analysis further validated the reliability of this model (Fig. S8). To further confirm the feasibility of the model, site-directed mutagenesis of amino acid residues surrounding the 2,3-oxidosqualene was performed in *Es*bAS1 and *Es*CAS1.

After site-directed mutagenesis of these amino acids either individually or in combination (Fig. S9), the results of the metabolite analysis indicated that all *Es*CAS1 and *Es*bAS1 mutants, except *Es*bAS1^W259L^, exhibited a complete loss of catalytic function, as no catalytic products were detected, similar to the null control (Fig. 4c). Interestingly, *Es*bAS1^W259L^ resulted in 50% reduction in β-amyrin production, which was associated with the formation of a new product, lupeol (Fig. 4c). Thus indicating that these amino acids (W259, Y261, W535, and F729 in *Es*bAS1 and W255, H257, Y532, F726, and N727 in *EsCAS1*) are critical for enabling 2,3-oxidosqualene to form a cyclized carbon chain structure.

### Functional analysis of *EsbAS1* and *EsCAS1* promoters

**Figure 5:**
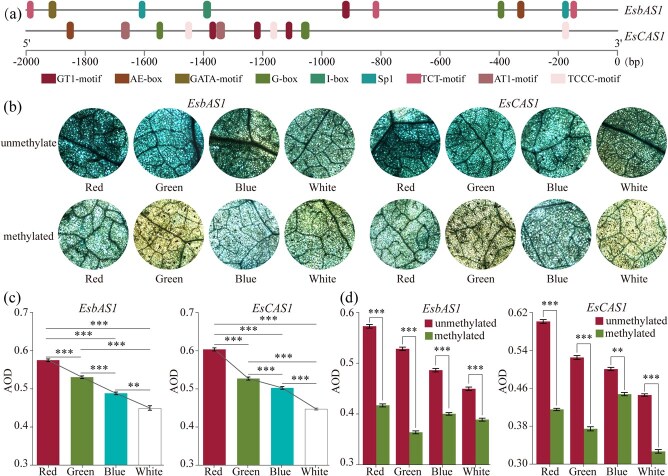
**Functional analysis of *EsbAS1* and *EsCAS1* promoters.** (**a**) Light-responsive elements in *EsbAS1* and *EsCAS1* promoters. (**b**) GUS staining of *N. benthamiana* leaves: staining effects of methylated and unmethylated promoters under growth in four different light quality, 4×. (**c**) Mean optical density values for GUS staining. (**d**) Effects of DNA methylation on *EsbAS1* and *EsCAS1* promoter activities (*P < 0.05; **P < 0.01; ***P < 0.001).

The promoters of *EsbAS1* and *EsCAS1* (Table S7) contained a diverse array of *cis*-acting elements (Fig. 1e) with the highest concentration of light-responsive elements (Figs 1e and 5a). This suggests that *EsbAS1* and *EsCAS1* play crucial roles in the response of *E. senticosus* to light changes that influence saponin synthesis.

GUS (β-glucuronidase) staining demonstrated that the promoters of both *EsbAS1* and *EsCAS1* could drive the expression of their respective genes (Fig. 5b). However, their initiation efficiencies varied under different colored light conditions. The *EsbAS1* and *EsCAS1* promoters exhibited the highest initiation efficiencies under red light, which were 1.28 and 1.35 times greater than those observed under white light, respectively. Overall, the initiation efficiencies decreased in the order of red, green, blue, and white light irradiation (Fig. 5b and c). DNA methylation resulted in a significant reduction in the initiation efficiencies of the *EsbAS1* and *EsCAS1* promoters (*P* < 0.001), decreasing to 68.89%–86.50% and 69.10%–89.41% of the efficiencies of the unmethylated promoters, respectively (Fig. 5b and d). This indicates that both light quality and DNA methylation significantly influenced the expression of *EsbAS1* and *EsCAS1* by altering the initiation efficiency of their promoters.

### Screening for transcription factors that bind to *EsbAS1* and *EsCAS1* promoters

**Figure 6:**
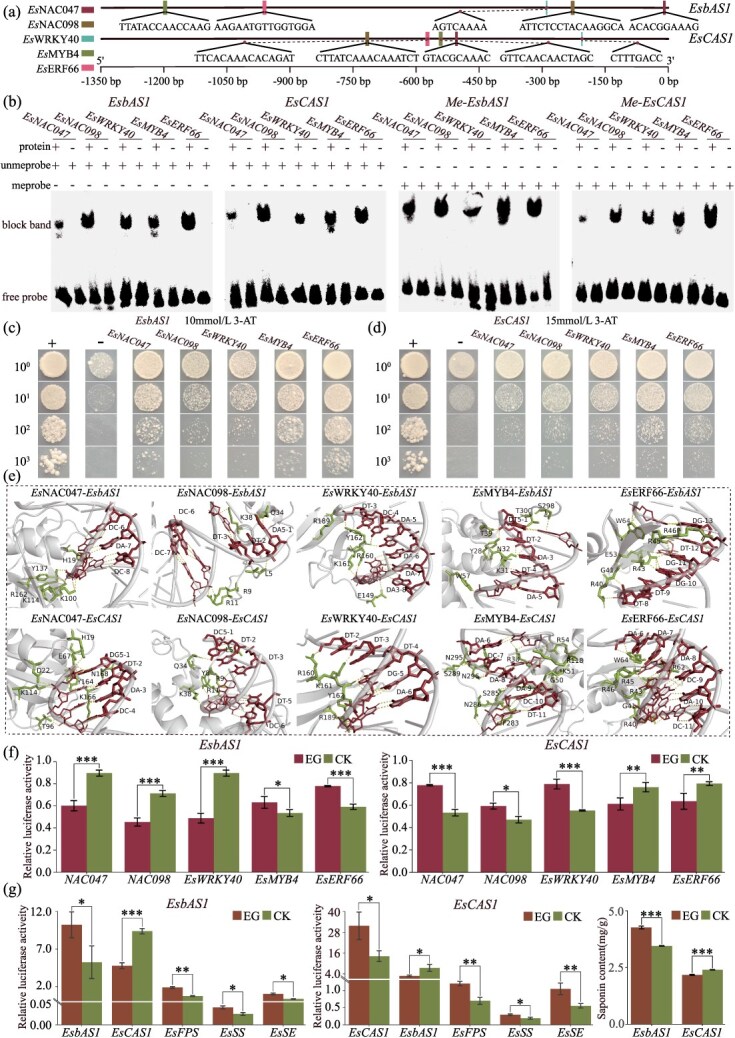
**Mechanism of transcription factor regulation of *EsbAS1* and *EsCAS1* promoters.** (**a**) Binding sites of transcription factors to *EsbAS1* and *EsCAS1* promoters. (**b**) EMSA analysis of transcription factor binding to *EsbAS1* and *EsCAS1* promoters. (**c**) Y1H analysis confirms binding of transcription factors to the *EsbAS1* promoter. (**d**) Y1H analysis confirms binding of transcription factors to the *EsCAS1* promoter. Transformed yeast cells grown on SD/−Trp/−Leu/–His dropout media. ‘+’ is pHis2-p53 + pGADT7-p53 positive control. ‘–’ is pHis2-pro*EsbAS1*/pro*EsCAS1* + pGADT7 negative control. (**e**) Molecular docking. (**f**) Dual luciferase reporter assay of transcription factors and *EsbAS1* and *EsCAS1* promoters. (**g**) Effect of *EsbAS1* and *EsCAS1* gene overexpression on *E. senticosus* saponin synthesis (*P < 0.05; **P < 0.01; ***P < 0.001), EG (experimental group), CK (control group).

The results predicted using JASPAR indicated that transcription factors *Es*NAC047, *Es*NAC098, *Es*WRKY40, *Es*MYB4, and *Es*ERF66 could bind to various sites in the promoters of *EsbAS1* and *EsCAS1* (Fig. 6a). Furthermore, an electrophoretic mobility shift assay (EMSA) was conducted using biotin-labeled *EsbAS1* and *EsCAS1* promoter probes along with the aforementioned transcription factors that were expressed and purified *in vitro*. The results demonstrated that the probes could bind to these five transcription factors, forming a complex irrespective of DNA methylation, resulting in the appearance of lagging bands in the corresponding lanes (Fig. 6b). This finding indicates that all these transcription factors can bind to the promoters of *EsbAS1* and *EsCAS1* and that this binding is unaffected by the DNA methylation status of these promoters.

To further verify whether the transcription factors bind to the promoters of *EsbAS1* and *EsCAS1*, a yeast one-hybrid (Y1H) assay was conducted. Yeast cells were diluted and plated in various concentration gradients (10^0^, 10^1^, 10^2^, 10^3^) and spot-coated on SD/−Trp/−Leu/–His dropout media supplemented with 0–15 mmol/l 3-AT. Following standard incubation, results indicated that the growth of the negative control (pHis2-pro*EsbAS1*/pro*EsCAS1* + pGADT7) was inhibited on media containing 10 and 15 mmol/l 3-AT, respectively. Consequently, the optimal 3-AT inhibitory concentration for the Y1H bait vector pHis2-pro*EsbAS1* was determined to be 10 mmol/l, while for pHis2-pro*EsCAS1*, it was 15 mmol/l (Fig. S10). The positive control group (pHis2-p53 + pGADT7-p53), negative control group, and experimental group (pHis2-pro*EsbAS1*/pro*EsCAS1*+ pGADT7-transcription factors) were spot-coated on 10 and 15 mmol/l 3-AT media. The results demonstrated that the growth of the negative control group (−) was inhibited, whereas the experimental group exhibited normal growth consistent with the growth status of the positive control group (+). The suggests that these transcription factors are capable of binding to the promoter regions of the *EsbAS1* and *EsCAS1* genes (Fig. 6c and d).

### Molecular docking of transcription factors with *EsbAS1* and *EsCAS1* promoters

Molecular docking analysis revealed that *Es*NAC047, *Es*NAC098, *Es*WRKY40, *Es*MYB4, and *Es*ERF66 can bind to the CAC, ATTCC, TCAAAA, TTATA, and TTGGTG nucleotides of the *EsbAS1* promoters with 5–7 amino acids. Additionally, these transcription factors can sequentially bind to the GTAC, CTTTC, TTTGA, ACAACT, and AAACAC nucleotides of the *EsCAS1* promoter through 4–9 amino acids (Fig. 6e). These nucleotides form hydrogen bonds with specific amino acids located in the grooves or pockets of transcription factors, facilitating their interaction with the target DNA. Furthermore, molecular dynamics analysis indicated that stable binding could be achieved between these transcription factors and their respective promoters (Figs S11 and S12).

### Effects of transcription factors on *EsbAS1* and *EsCAS1* promoter activity

The results of the dual-luciferase reporter assay (Fig. 6f) demonstrated that the binding of *Es*NAC047, *Es*NAC098, and *Es*WRKY40 significantly reduced the activity of the *EsbAS1* promoter (*P* < 0.001). In contrast, *EsCAS1* promoter activity was significantly increased by these three transcription factors (*P* < 0.001 or *P* < 0.05). Conversely, the activity of the *EsbAS1* promoter was significantly increased (*P* < 0.001 or *P* < 0.05) by binding *Es*MYB4 and *Es*ERF66, whereas that of the *EsCAS1* promoter was significantly decreased (*P* < 0.01) by binding these two transcription factors.

### Overexpression of *EsbAS1* and *EsCAS1*

Compared with the empty vector control, the expression levels of both *EsbAS1* and *EsCAS1* were significantly increased after overexpression (*P* < 0.05), indicating that overexpression of *EsbAS1* and *EsCAS1* in *E. senticosus* was successfully achieved (Fig. 6g). Overexpression of *EsbAS1* suppressed the expression of *EsCAS1*. Similarly, overexpression of *EsCAS1* suppressed the expression of *EsbAS1* (*P* < 0.05). However, the overexpression of both significantly increased the expression of upstream saponin synthase genes (*EsFPS*, *EsSS*, and *EsSE*) (*P* < 0.05), suggesting significant competition for substrates between the two. Additionally, overexpression of *EsbAS1* significantly increased total saponin content in *E. senticosus* (*P* < 0.001), whereas overexpression of *EsCAS1* significantly decreased total saponin content (*P* < 0.001) (Fig. 6g).

## Discussion

*Eleutherococcus senticosus* contains several types of pentacyclic triterpene saponins, with oleanane-type saponins being the predominant component responsible for its medicinal effect. In contrast, other types of saponins, such as lupinane-type saponins, are present at extremely low concentrations [21] and their pharmacological effects differ significantly from those of oleanane-type saponins [22]. In addition, sterols with tetracyclic skeletons are essential for the growth and development of *E. senticosus* [17]. It has been demonstrated that the diversity of OSC underlies variations in triterpenoid saponin biosynthesis [19]. Various types of saponins and sterols are synthesized by the corresponding OSCs that catalyze their formation [12, 16, 17, 23]. Among these, bAS is the only OSC identified to date that catalyzes the biosynthesis of oleanane-type triterpenoid saponins, whereas CAS is responsible for sterol synthesis [24].

Consequently, *Es*bAS and *Es*CAS family members are pivotal in the metabolic pathways of saponins and sterols, influencing the content and structural diversity of triterpenoid saponins in *E. senticosus* [16, 17]. Essentially, *Es*CAS and *Es*bAS compete for substrates, and their gene expression regulates the ratio of 2,3-oxidosqualene entering the triterpene saponin metabolic pathway. Therefore, identification and functional analysis of *EsbAS* and *EsCAS* are crucial for understanding the mechanisms underlying saponin synthesis in *E. senticosus*.

Dozens of OSCs exist in nature that can catalyze the cyclization of 2,3-oxidosqualene into sterols and >100 triterpenoids with varying carbon skeletons [10, 25]. However, for a given species, the number of OSC family members is ~10 in most plants [25], except for the hexaploid *Triticum aestivum* [26]. The genome of *E. senticosus* contains 10 OSCs, which is essentially the same as that found in other diploid plants. Phylogenetic analyses revealed that the 10 *EsOSC*s could be classified into three branches: β-amyrin, cycloartenol, and lupeol. The β-amyrin branch contained the highest number of genes, whereas the lupeol branch contained only two genes. This observation aligns with the fact that oleanane-type saponins are the predominant saponins in *E. senticosus*, whereas other types of saponins are quite rare [21, 27]. The *EsOSC* gene family exhibits distinct exon–intron distribution patterns. The β-amyrin synthase branches of *EsOSC4, 5, 6, 7*, and *8* shared similar structures, with exons concentrated in the latter half of the gene and were the most abundant. The cycloartenol synthase branch, comprising *EsOSC2, 3*, and *10*, exhibited similar structures, albeit with relatively few exons. This suggests that genes within the same evolutionary branch may have evolved from a common ancestor and that a higher exon count may indicate more complex gene structures, potentially resulting from events such as selective splicing or gene duplication [28]. The conserved structural domains of *Es*OSC comprise nearly full-length protein sequences, which align perfectly with the characterization of the conserved structural domains of OSC from the N-terminus to the C-terminus in species such as *Camellia sinensis* [29]. *Es*OSC contains 2–6 conserved motifs, including the DCTAE motif (motif 3), which is involved in substrate binding, MWCYCR motif (motif 4), and two QXXXXXW repeats. These motifs have also been associated with the stabilization of carbon cation intermediates during the cyclization of 2,3-oxidosqualene [30] (Fig. S2).

Enzymes with similar functions typically exhibit comparable structures [31]. Phylogenetic analyses revealed that *EsOSC6* and *EsOSC2* clustered with the bAS and CAS of other species, respectively. The expression of *EsOSC6* was positively correlated with genes and metabolites involved in saponin synthesis, whereas the expression of *EsOSC2* was negatively correlated. These correlation profiles were perfectly aligned with the *Pg*bAS of *P. ginseng* and the *Ws*CAS profiles of *W. somnifera* [17, 18]. This suggests that *EsOSC6* functions as *Es*bAS1, catalyzing the synthesis of saponins, whereas *EsOSC2* acts as *Es*CAS1, catalyzing the synthesis of sterols.

Studies have demonstrated that OSCs primarily function in the cytoplasm, with *Bc*BAS1 from *Bupleurum chinense* localized in the cytoplasmic lysate [32], and *Sg*CAS from *Siraitia grosvenorii* found in either the nucleus or cytoplasm [33]. Similarly, this study revealed that *Es*bAS1 and *Es*CAS1 were predominantly located in the cytoplasm.

The low synthetic capacity of 2,3-oxidosqualene in natural *Saccharomyces cerevisiae* poses challenges for subsequent analyses of OSC functions. Consequently, *S. cerevisiae* strains genetically modified to synthesize 2,3-oxidosqualene are frequently used in OSC functional studies [34]. In this study, among the αAM2 strains capable of producing adequate 2,3-oxidosqualene, *Es*bAS1 was identified exclusively as a β-amyrin synthase, whereas *Es*CAS1 was identified exclusively as a cycloartenol synthase, corroborating previous predictions. Notably, the quantity of cycloartenol catalytically produced by *Es*CAS1 was significantly lower than that produced by *Es*bAS1, and no residues of the substrate 2,3-oxidosqualene were detected in either the control or any of the experimental groups analyzed, suggesting complete utilization of the substrate. Based on previous reports indicating that *S. cerevisiae* utilizes its 2,3-oxidosqualene to produce lanosterol [35], it was hypothesized that *Es*bAS1 and *Es*CAS1 would compete with αAM2 for the substrate 2,3-oxidosqualene, with *Es*CAS1 being less competitive, resulting in a low yield of cycloartenol [36]. In addition, other peaks were also detected, which may represent metabolites modified by *S. cerevisiae* [37].

The activities of both *Es*bAS1 and *Es*CAS1 were characterized by high efficiency, with the yields of the corresponding products stabilizing as the catalytic time increased. Certain key amino acid residues significantly influence the catalytic outcomes of OSC, resulting in variations in their products [38, 39]. The differentiation and evolution of OSC have led to considerable sequence diversity and functional plasticity, and the catalytic mechanism of bAS has been studied extensively [40]. This study revealed that the catalytic center of most plants contains a highly conserved MWCYCR motif, whereas the lupeol synthases of pentacyclic triterpene alcohols exhibit an almost identically conserved MLCYCR motif. The W259L mutation in the MWCYCR motif of *Pg*bAS is sufficient to reprogram the enzyme to produce lupeol as the primary product [41]. Additionally, the L253W substitution in the MLCYCR motif of lupeol synthases from *Perilla frutescens* leads to the production of substantial amounts of β-amyrin [42]. In this study, the mutation sites involved the conserved MWCYCR motifs (W259 and Y261). The *Es*bAS1^W259L^ mutant shifted from a single product β-amyrin to an equal mixture of β-amyrin and lupeol. The *Es*bAS1^Y261H^ mutant lost its enzymatic function, exemplifying the functional plasticity of *Es*bAS1. Mutations in corresponding residues, such as W259L in bAS from *C. sinensis* and W257L and Y261H in bAS from *P. frutescens* [29, 42], exhibited similar effects. Multiple sequence comparisons indicated that *Es*CAS1 possesses nearly identical conserved MWCHCR motifs, and *Es*CAS1^W255L^ and *Es*CAS1^H257Y^ mutants demonstrated no product generation and comparable loss of enzymatic function.

Further molecular docking analyses revealed that W535 was located near the MWCYCR motif in the upper notch of the adjacent substrate, whereas Y532 was located near the MWCHCR motif. Multiple sequence comparisons indicated that both amino acid sites were equally conserved and no product generation was observed for *Es*bAS1^W535Y^ and *Es*CAS1^Y532W^. It has been demonstrated that the active center of the OSC is characterized by a highly conserved W-xY triplex that is spatially clustered. For instance, the *Gg*bAS enzyme from *Glycyrrhiza glabra* features the classical W534-W257Y259 triplex, whereas *It*OSC3 from *Iris tectorum* contains the Y534-WH259 triplet [20]. The triple mutants *Es*bAS1 (W535Y/W259L/Y261H) and the triple mutant of *Es*CAS1 (Y532W/W255L/H257Y) exhibited a similar loss of enzymatic function, suggesting that the triplex is highly conserved and represents an essential site for OSC activity. In summary, the active centers of *Es*bAS1 and *Es*CAS1 correspond to W(535)-WY and Y(532)-WH triplets, respectively.

The abundance of light-responsive elements in the *EsOSC* promoter suggests that *EsOSC* is a light-responsive gene. When exposed to various colored light wavelengths, the promoters of *EsbAS1* and *EsCAS1* were able to drive the expression of GUS to varying extents. This further demonstrates that the promoters of *EsbAS1* and *EsCAS1* can modulate their expression in response to changes in light quality, thereby influencing the synthesis of triterpenoid saponins, similar to the behavior observed with other saponin synthase gene promoters [7]. Studies have shown that DNA methylation can significantly inhibit the activity of most gene promoters [6, 14]. In this study, the activity of *EsbAS1* and *EsCAS1* promoters was also significantly reduced in the presence of DNA methylation.

Alterations in gene expression primarily result from indirect changes in target gene expression, which are influenced by variations in transcription factor expression [43]. Transcription factors typically possess the ability to bind directly to the promoters of downstream target genes, thereby regulating their expression [7, 14, 44]. For instance, *Es*bZIP and *Es*NAC transcription factors can directly interact with the promoters of *EsFPS*, *EsSS*, and *EsSE*, thereby modulating their expression [6, 7]. In this study, EMSA, Y1H, and molecular docking analyses further confirmed that the five transcription factors can stably bind to the promoters of *EsbAS1* and *EsCAS1*, which subsequently activate or repress gene expression.

Transcription factors regulate the expression of various genes. *Cs*MYB123 from *Chaenomeles speciosa* can bind to the promoters of structural genes associated with anthocyanin biosynthesis, thereby positively influencing its activity [45]. *Ps*ERF1B and *Ps*MYB10.1 of *Prunus salicina*, both of which function as positive regulators, can individually enhance the activity of the *PsUFGT* promoter, leading to upregulation of gene expression [46]. In this study, *Es*MYB4 and *Es*ERF66 positively regulated *EsbAS1* expression and negatively regulated the expression of *EsCAS1*. Conversely, *Es*NAC047, *Es*NAC098, and *Es*WRKY40 regulated *EsbAS1* and *EsCAS1* in opposing manners. Notably, the ability of these transcription factors to regulate the expression of *EsbAS1* and *EsCAS1*, genes that perform distinct functions, in completely opposite ways has not been documented in other species. Furthermore, this opposing regulation creates favorable conditions for the quality enhancement of *E. senticosus*.

Transient overexpression of *EsbAS1* and *EsCAS1* resulted in an increase or decrease in oleanane-type saponin content in *E. senticosus*. The expression levels of key enzyme genes involved in the synthesis of 2,3-oxidosqualene (*EsFPS*, *EsSS*, and *EsSE*) were significantly elevated. This suggests that *Es*bAS1 and *Es*CAS1 compete for the substrate 2,3-oxidosqualene, and their expression exhibits a reciprocal relationship, which aligns with the regulation of transcription factors identified in this study (Fig. 6d). Therefore, manipulating the expression of the relevant transcription factors to regulate *EsbAS1* and *EsCAS1* could enhance the quality of *E. senticosus*.

## Conclusion

**Figure 7:**
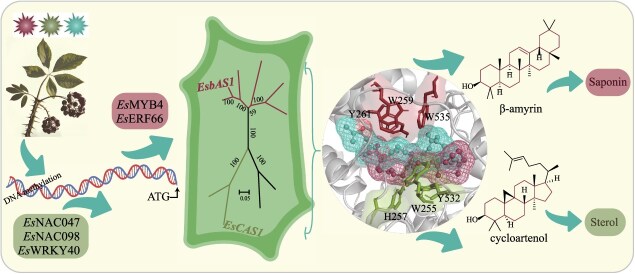
Mechanism of *EsOSC* regulation of *E. senticosus* saponin synthesis.

In summary, we preliminarily screened *EsOSCs* associated with saponin and sterol synthesis in *E. senticosus* using genome-wide identification and correlation analyses. Catalytic assays confirmed that *Es*bAS1 functions as a β-amyrin synthase, exclusively catalyzing the formation of β-amyrin, whereas *Es*CAS1 acts as a cycloartenol synthase, exclusively catalyzing the formation of cycloartenol. *Es*bAS1 and *Es*CAS1 primarily operate in the cytoplasm and their active sites are located in the W-WY and Y-WH triplets, respectively. Additionally, *EsbAS1* and *EsCAS1* possess light-dependent promoters and their initiation efficiency is influenced by light quality and DNA methylation. Finally, we identified five transcription factors that regulated *EsbAS1* and *EsCAS1* expression (Fig. 7). Therefore, this study establishes a foundation for comprehensive analysis of the molecular mechanisms underlying saponin synthesis in *E. senticosus*.

## Materials and methods

### Plant material and reference standards

In this study, 2-year-old *E. senticosus* plants propagated from cuttings were used as experimental material. Leaves from various growth and developmental stages were selected for RNA extraction and saponin content analyses. The reference standards used were β-amyrin, cycloartenol (Alfa Biotechnology, China), lupeol, and lanosterol (Aladdin Biochemical Technology, China).

### Identification and analysis of *EsOSC* gene family

The *AtOSC* gene of *Arabidopsis thaliana* (www.arabidopsis.org/) was used as the reference gene to identify all *EsOSCs* from the genome sequencing data of *E. senticosus* [5]. The Hidden Markov Model of OSC (PF13243, PF13249) from the PFAM database served as the standard for further verification of the *EsOSCs*. The physicochemical properties, subcellular localization, and conserved structural domains of all *EsOSCs* were analyzed in accordance with established methods [14, 15, 47]. Phylogenetic analysis of OSC was conducted using MEGA 11 software and the *EsOSC* motif was examined using MEME software. Additionally, *cis*-acting elements of *EsOSC* gene promoters were analyzed using PLANTCARE [14, 15].

### Expression analysis and correlation analysis of *EsOSCs*

The expression levels of all *EsOSC* and other triterpenoid synthase genes were analyzed using transcriptome sequencing data from *E. senticosus* exposed to different light irradiation colors [7]. In addition, information regarding saponin metabolite content was obtained from the metabolome analysis data of the corresponding samples. The correlation between the expression of *EsOSC* and the *E. senticosus* triterpenoid saponin synthase gene and metabolite content was analyzed using the SPSS 26.0 software [6].

### Quantitative reverse transcription polymerase chain reaction analysis of *EsOSCs* and determination of saponin content

The glyceraldehyde-3-phosphate dehydrogenase (GAPDH) gene served as an internal reference gene [9, 48]. RNA was extracted from each sample of *E. senticosus* following a previously established method and reverse-transcribed into complementary DNA. *EsOSC* expression was quantified using quantitative reverse transcription polymerase chain reaction (qRT-PCR) [7, 14]. The primers used in this study are listed in Table S1. Additionally, the total saponin content of *E. senticosus* was determined using high-performance liquid chromatography (HPLC) [9].

### Subcellular localization of *Es*bAS1 and *Es*CAS1

Primers were designed based on the nucleotide sequence of *EsOSC* and the ORFs of *EsbAS1* and *EsCAS1* were obtained through PCR amplification (Table S1). Primers containing homology arms were designed separately (Table S1), and the ORFs of *EsbAS1* and *EsCAS1* were inserted into PHG expression vectors using a Seamless Cloning Kit (Beyotime, China). The recombinant vector was successfully constructed and transformed into the GV3101 strain of *Agrobacterium tumefaciens*, and the abaxial surface of *N. benthamiana* leaves was infiltrated with *A. tumefaciens* for expression in *N. benthamiana*. Two days later, the infiltrated area was cut and sectioned, and observed with a laser scanning confocal microscope.

### Heterologous expression of *EsbAS1* and *EsCAS1*

Heterologous expression vectors *EsbAS1*-pESC-Trp and *EsCAS1*-pESC-Trp were constructed as previously described. Primers were designed according to the instructions provided in the M5 HiPer Site-Directed Mutagenesis Kit manual (Table S1) (Mei5 Biotechnology Co., Ltd., China). The conserved bases of *Es*bAS1 and *Es*CAS1 were subjected to site-directed mutagenesis and the resulting mutated *EsbAS1* and *EsCAS1* were subsequently inserted into the heterologous expression vector pESC-Trp. The recombinant vectors were transformed into the αAM2 strain of *S. cerevisiae* and expressed in SD/−Trp broth.

### Analysis of catalytic activities of *Es*bAS1 and *Es*CAS1

αAM2 metabolites were extracted from the αAM2 strain obtained in the previous step after 12–84 h of induced incubation, following the method described by Sandeep *et al* [49]. β-Amyrin and cycloartenol metabolites were determined using the LabSolutions HPLC system (Shimadzu, Japan). The detection parameters were as follows: column temperature, 35°C; mobile phase, 1:1 mixture of methanol and acetonitrile; flow rate, 0.8 ml/min; detection wavelength, 203 nm; and total analysis time, 80 min. The β-amyrin content was calculated using the standard curve represented by the equation y = 10106039.06x–10471.45, which was determined using the standard curve given by the equation y = 8518.47x + 2765.95, where y represents the peak area, and x denotes the concentration.

### DNA methylation

The *Es*C5-MTase protein was incubated for 1 h at 37°C in a 20-μl reaction containing 20 mM Tris (pH = 8.0), 1 mM EDTA, 2 mM DTT, 0.32 nM S-adenosylmethanethionine, 1 μg DNA, and 2 μM EsC5-Mtase [15].

### *EsbAS1* and *EsCAS1* promoter cloning and GUS staining

*EsbAS1* and *EsCAS1* promoter sequences were obtained by PCR amplification using specific primers (Table S1). The promoters were subsequently inserted into the pCAMBIA1304 vector via homologous recombination. The recombinant vector was then infiltrated into *N. benthamiana* using the GV3101 strain of *A. tumefaciens* [15]. Based on the results of *cis*-acting element analysis, *N. benthamiana* was incubated under red, green, blue, and white light for 3 days [7]. Staining was performed using the GUS Staining Kit (Coolaber, China) [14].

### Screening for transcription factors

Using the JASPAR database (jaspar.elixir.no/), we analyzed the ability of cloned transcription factors from *E. senticosus* to bind to the *EsbAS1* and *EsCAS1* promoters. We screened for transcription factors *Es*NAC047, *Es*NAC098, *Es*WRKY40, *Es*MYB4, and *Es*ERF66 that may interact with *EsbAS1* and *EsCAS1* promoters [6, 7, 14].

### EMSA

The ORF of the transcription factors identified in the previous step was inserted into the PGEX-4 T-3 plasmid using the homologous recombination method, as described previously. This construct was then transferred into *Escherichia coli* BL21 (DE3) cells to induce protein expression [47]. Transcription factor proteins were purified using a GST-tag protein purification kit (Beyotime Biotechnology, China) [50]. Based on the binding sites of the transcription factors, the promoters of *EsbAS1* and *EsCAS1* were used as templates, and DNA probes for the promoters (Table S2) were generated by PCR amplification using the primers listed in Table S1. The purified transcription factor proteins were then subjected to an EMSA-binding reaction with biotin-labeled normal and methylated DNA probes, following the method of Dong *et al*. [14].

### Y1H assay

The ORFs of transcription factors *Es*NAC047, *Es*NAC098, *Es*WRKY40, *Es*MYB4, and *Es*ERF66 were inserted into the pGADT7 to generate the prey vector. The promoters of *EsbAS1* and *EsCAS1* was inserted into the pHis2 to generate the bait vector. The primers are listed in Table S1. Subsequently, the bait vectors were combined two by two in prey vectors and co-transfected into Y187 yeast cells and the gradient-diluted bacterial fluids were spot-coated on SD/−Trp/−Leu/–His dropout media and grown for 3–4 days. pHis2–53 + pGADT7–53, pGADT7 vectors with the corresponding bait vectors were respectively used as a positive and negative control [51].

### Dual-luciferase reporter assay

Using homologous recombination, the ORF of each transcription factor was ligated into the pGreen II 62-SK vector to serve as an effector plasmid. The promoters of *EsbAS1* and *EsCAS1* were then inserted into the pGreen II 0800-LUC vector, which functioned as a reporter plasmid. The recombinant plasmid was transferred into *A. tumefaciens* GV3101 and infected with *N. benthamiana*. The impact of transcription factor binding to *EsbAS1* and *EsCAS1* promoters on promoter activity was analyzed 3 days later using a dual-luciferase reporter gene assay kit (Beyotime Biotechnology, China) [7, 14].

### Overexpression of *EsbAS1* and *EsCAS1*

The ORFs of *EsbAS1* and *EsCAS1* were inserted into the overexpression vector, pCAMBIA1300. The recombinant plasmid was transferred into *A. tumefaciens* strain GV3101 and *E. senticosus* leaves were infested. After 3 days of incubation, the expression levels of *EsbAS1* and *EsCAS1*, as well as the total saponin content, were analyzed by qRT-PCR and HPLC [7].

### Molecular docking and molecular dynamics simulations

Homology models of *EsbAS1* and *EsCAS1* were constructed using the SWISS-MODE website (Swissmodel.expasy.org/) using A8CDT2.1.A and A0A5J4ZY65.1.A as templates. The structures of the 2,3-oxidosqualene compounds were obtained from PubChem and optimized to the minimum energy state by Chem3D. Molecular docking of *Es*OSC with 2,3-oxidosqualene, along with the promoters of *Es*OSC and transcription factors, was performed using the AutoDock Vina and HDOCK Server databases [15]. Molecular dynamics simulations were performed with the GROMACS 2023.2 using the CHARMM36 and AMBER force fields [52]. The ligand and protein need to be parameterized separately and the base coordinates of the ligand are assigned according to the positions recorded in the RCSB. The protein–ligand complexes were placed in the box after charge balancing ions containing water molecules. Before the start of the simulation, perform energy minimization and Nvt, Npt pre-equilibrium 10^6^ times. Finally, the molecular dynamics simulations were run 5 × 10^7^ times, all simulations using a time length of 2 fs.

### Statistical analysis

The experiments were conducted in a randomized design with at least three individual replicates in each group. Statistical significance were analyzed using independent samples *t*-test or one-way ANOVA by SPSS 20.0, *P* < 0.05. All resultant data were plotted as graphs using GraphPad Prism 10.

## Supplementary Material

Web_Material_uhaf133

## Data Availability

All data in this study were provided in the article and its supplementary materials.
