# Supplementary material for: Identification of 2,3-oxidosqualene cyclase gene in Eleutherococcus senticosus and its regulatory mechanism in saponin synthesis
Source: Hortic Res. 2025 May 21;12(8):uhaf133. doi: 10.1093/hr/uhaf133 (PMC12268154; doi:10.1093/hr/uhaf133)
Supplement: Web_Material_uhaf133 [file web_material_uhaf133.zip › Supplementary figure.pdf]

The following Supporting Information is available for this article:

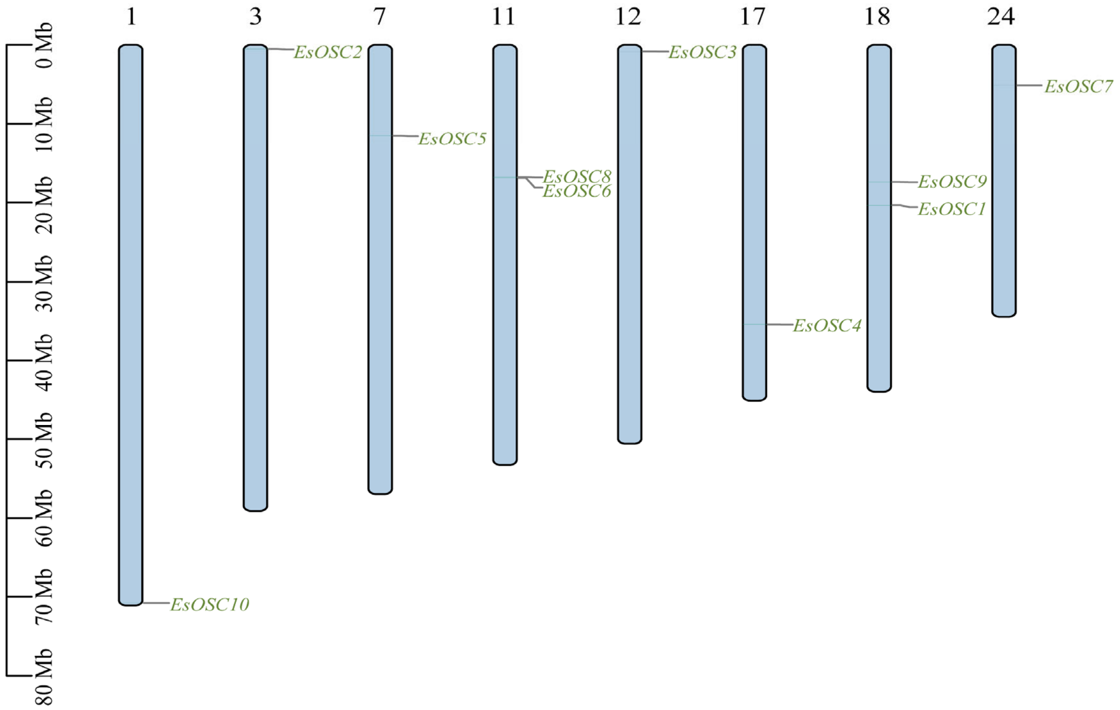

Figure S1 The location of *EsOSC* gene on chromosome.

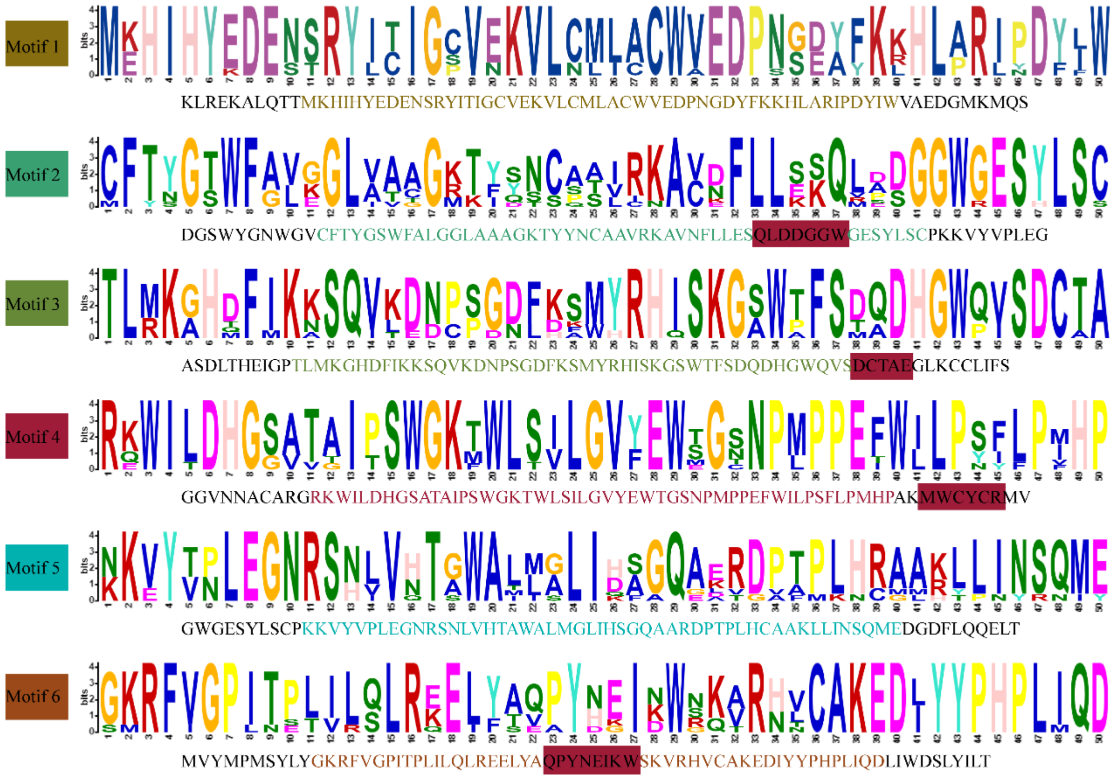

Figure S2 Motif compositions of *EsOSC*.

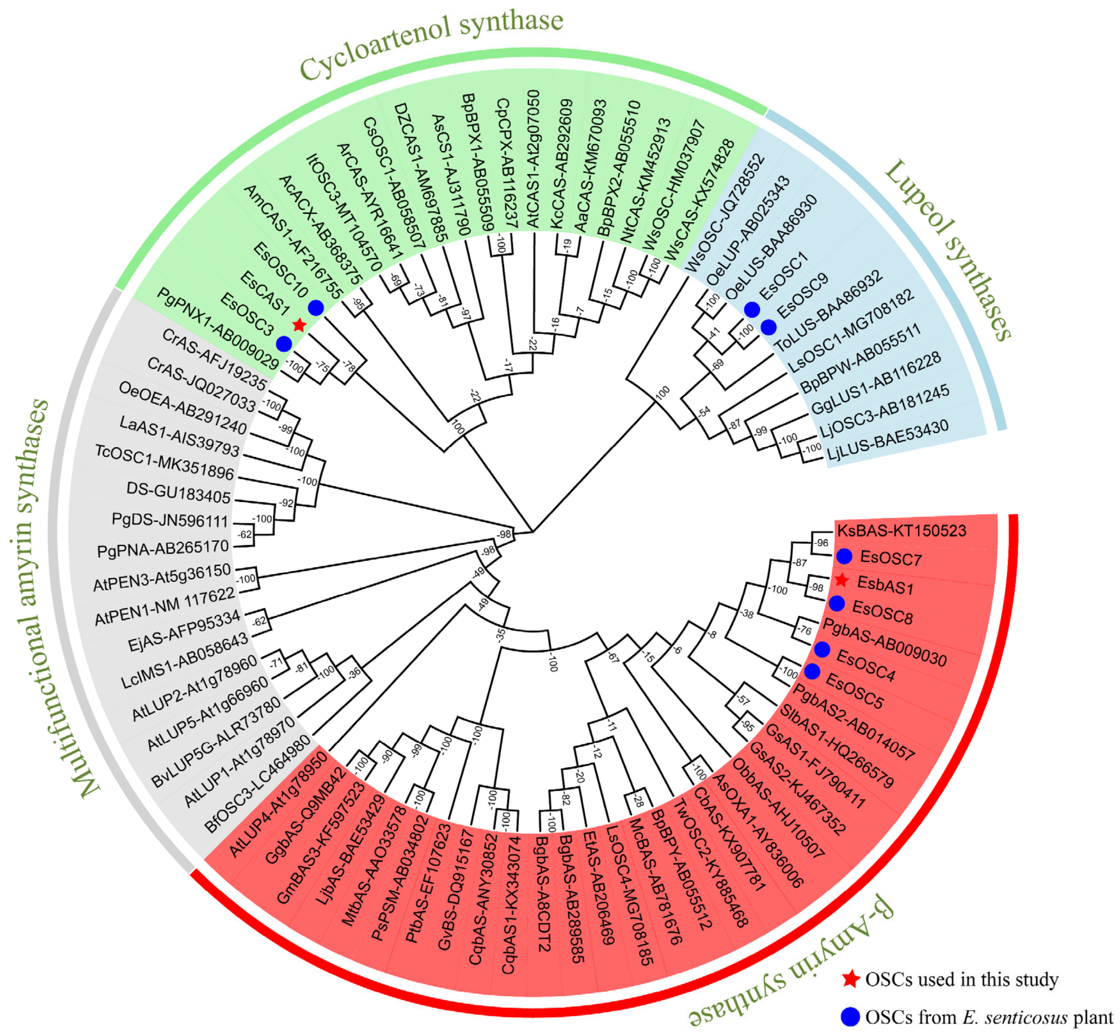

Figure S3 Phylogenetic analysis of *Es*OSC transcription factors.

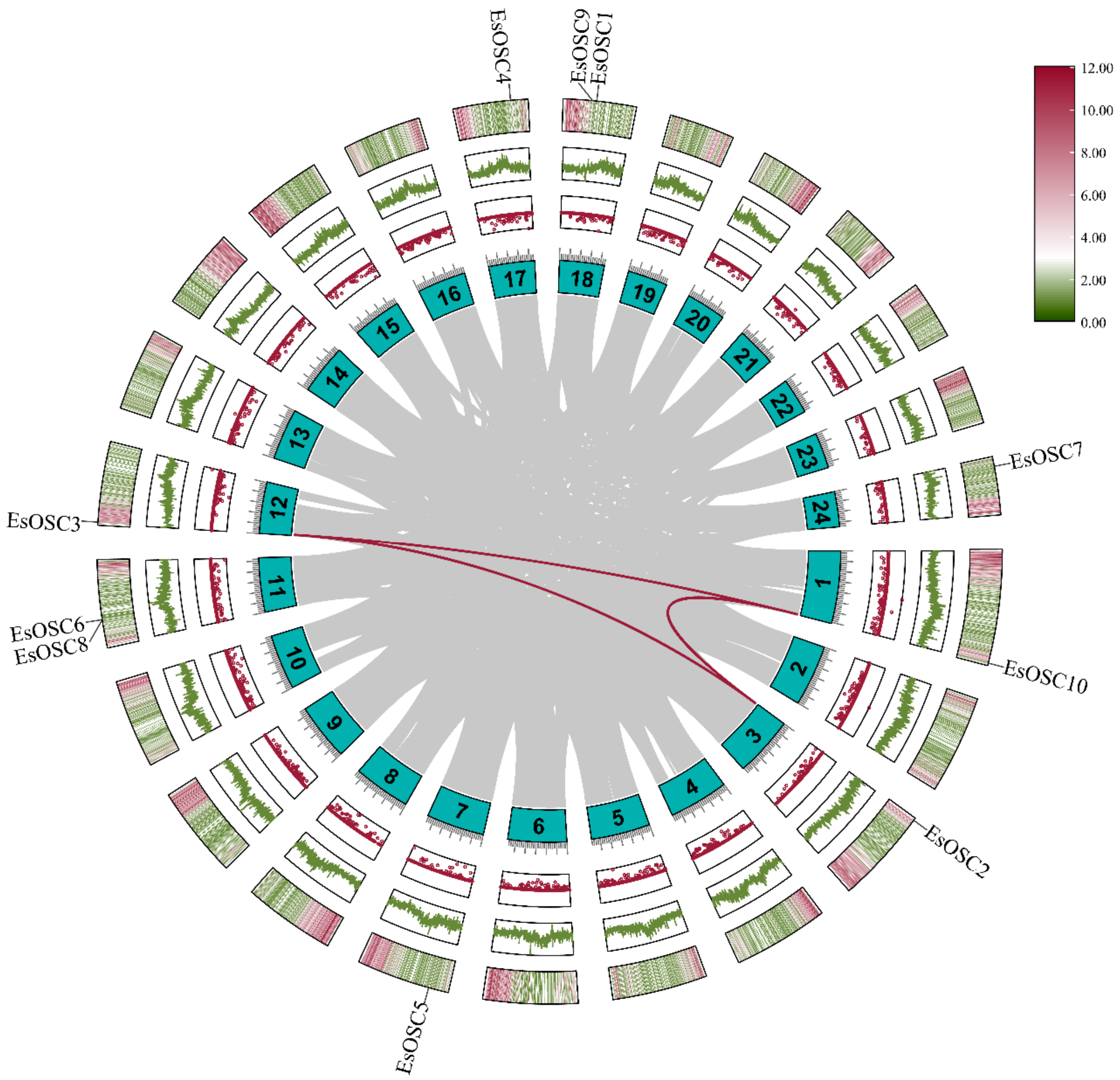

Figure S4 Intraspecific collinearity analysis of *EsOSC*. The numbers represent chromosomes.

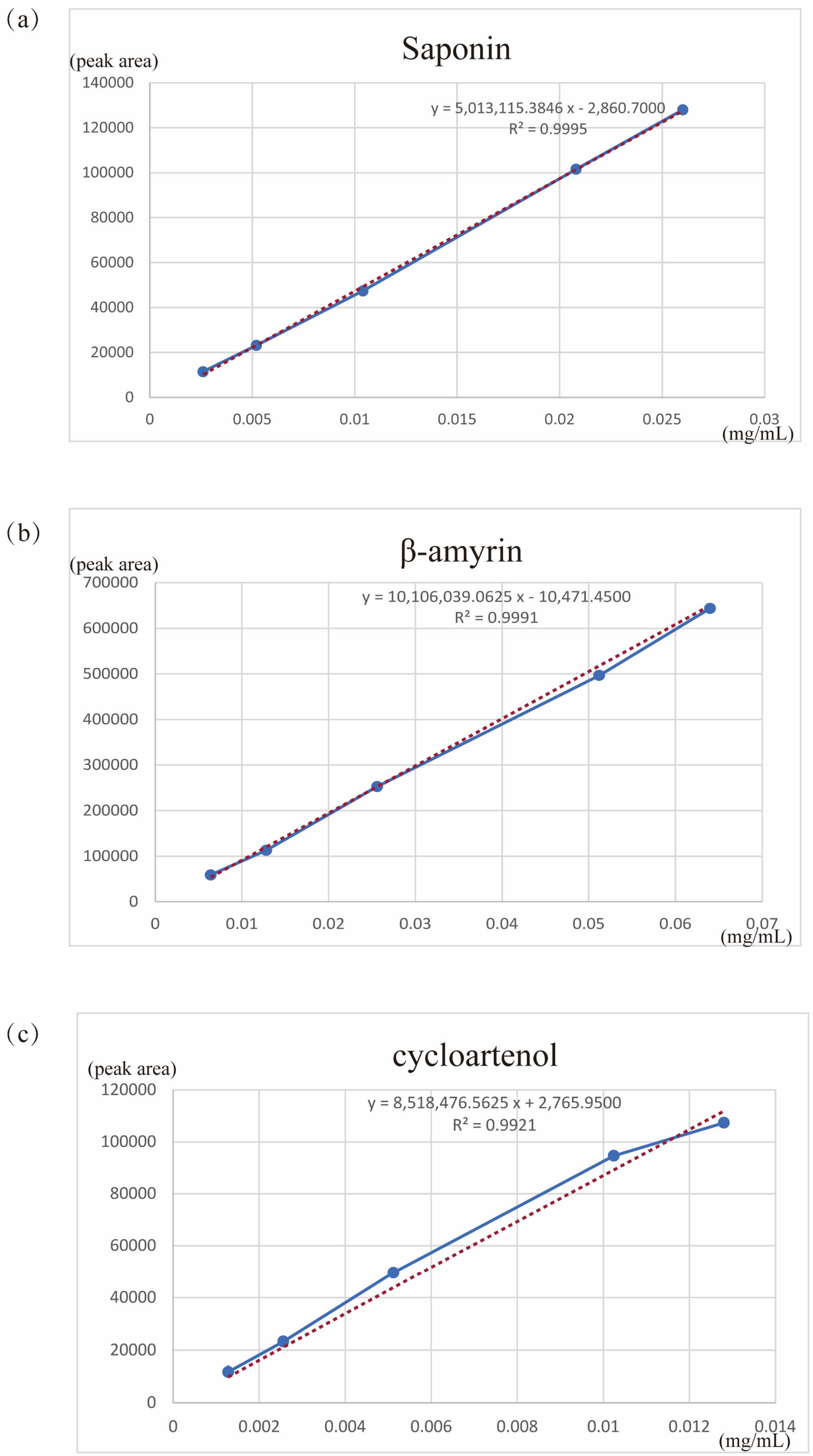

Figure S5 Standard curve for compounds.(a) Standard curve of saponin.(b) Standard curve of β-amyrin.(c) Standard curve of cycloartenol.

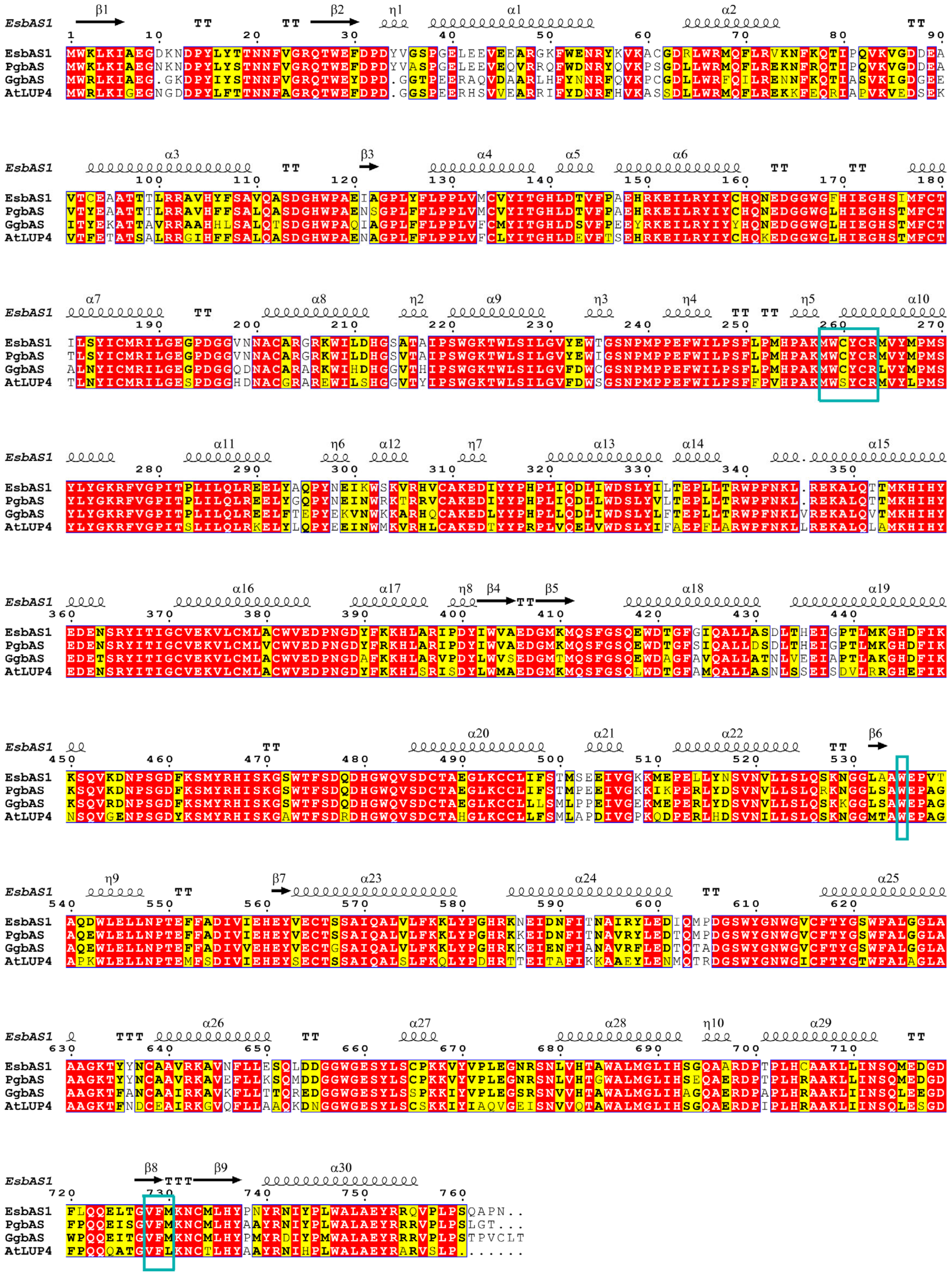

Figure S6 Multiple sequence alignment of *Es*bAS1 in *E. senticosus* and OSCs in other plants. The blue box shows the conserved amino acid motif.

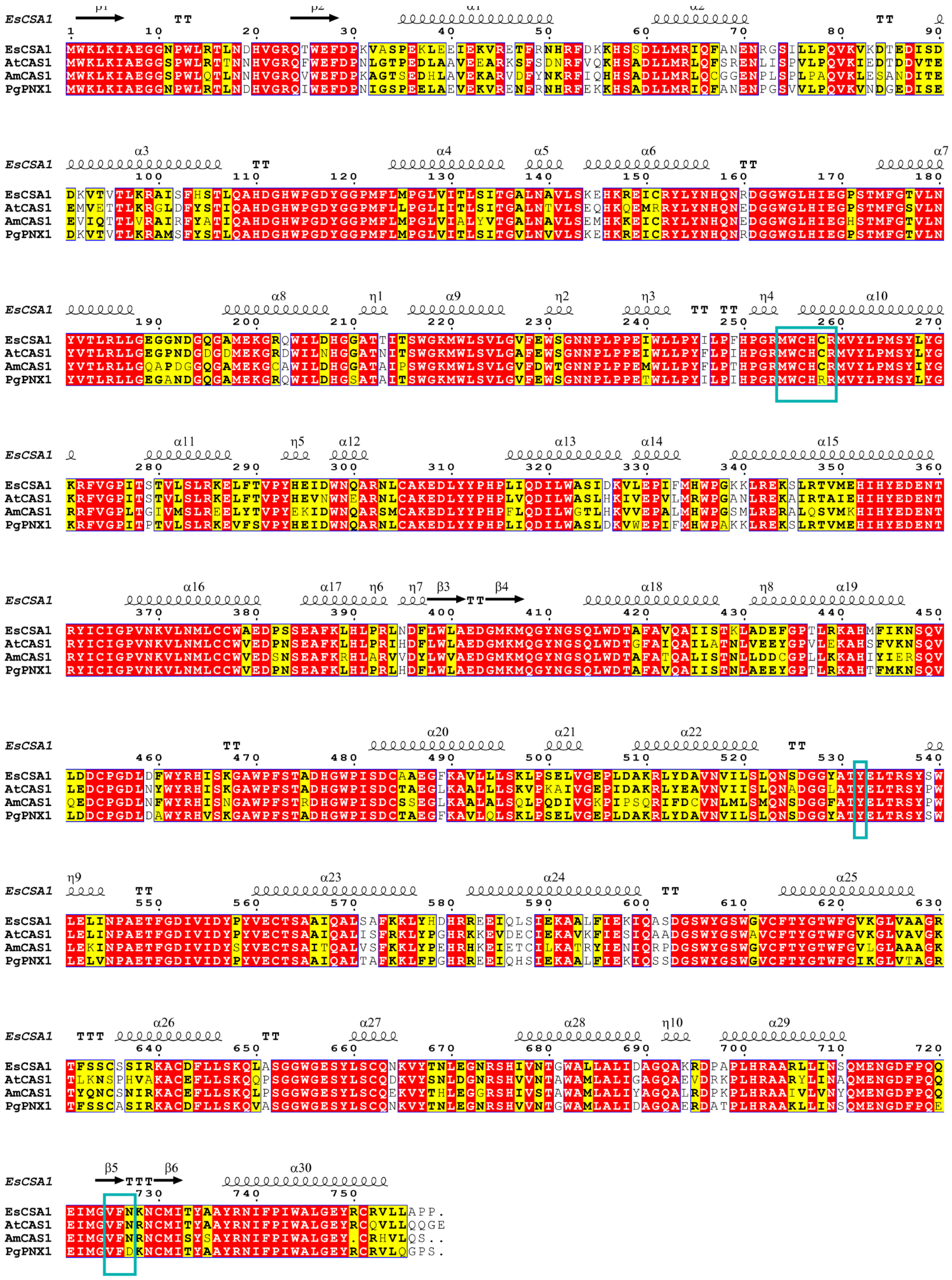

Figure S7 Multiple sequence alignment of *Es*CAS1 in *E. senticosus* and OSCs in other plants. The blue box shows the conserved amino acid motif.

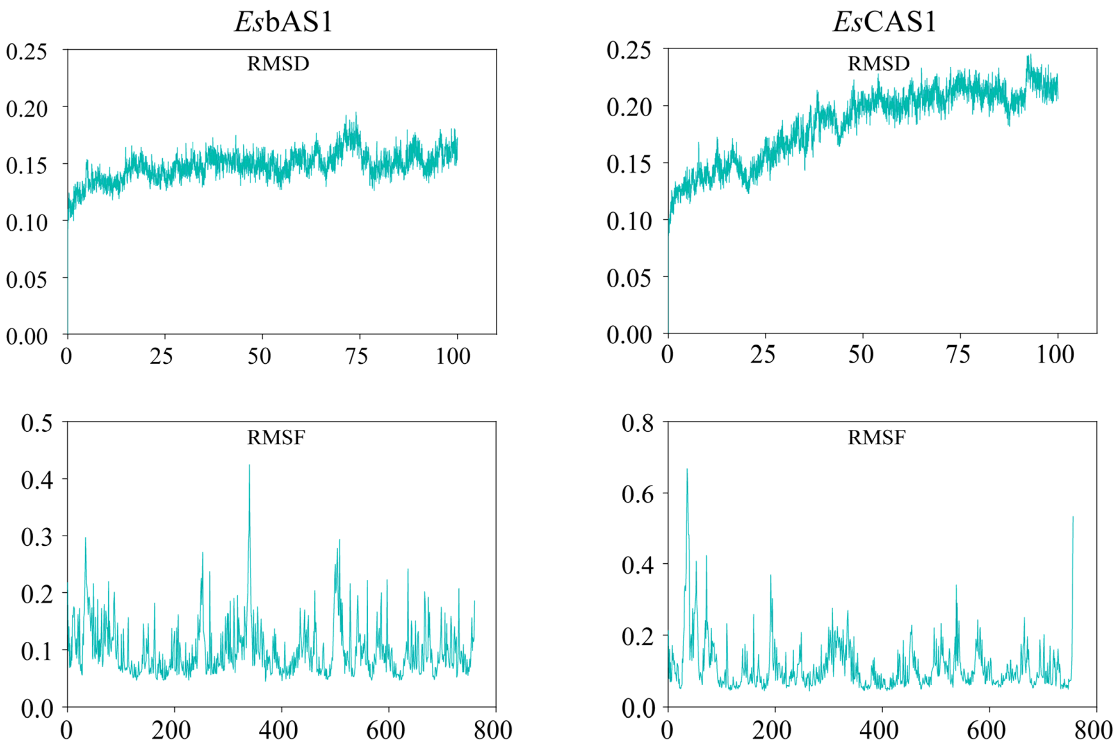

Figure S8 Results of molecular dynamics simulations of *Es*bAS1 and *Es*CAS1 with 2, 3-oxidosqualene. The root mean square deviation (RMSD) was performed to assess differences and changes in structure and evaluate the stability of the complexes. Root mean square fluctuation (RMSF) was used to analyze the movement of amino acids. The RMSD fluctuation range was 0.25A, and the RMSF fluctuation range was 0.1-0.8A. These results provide further evidence of the stability of the molecular docking model.

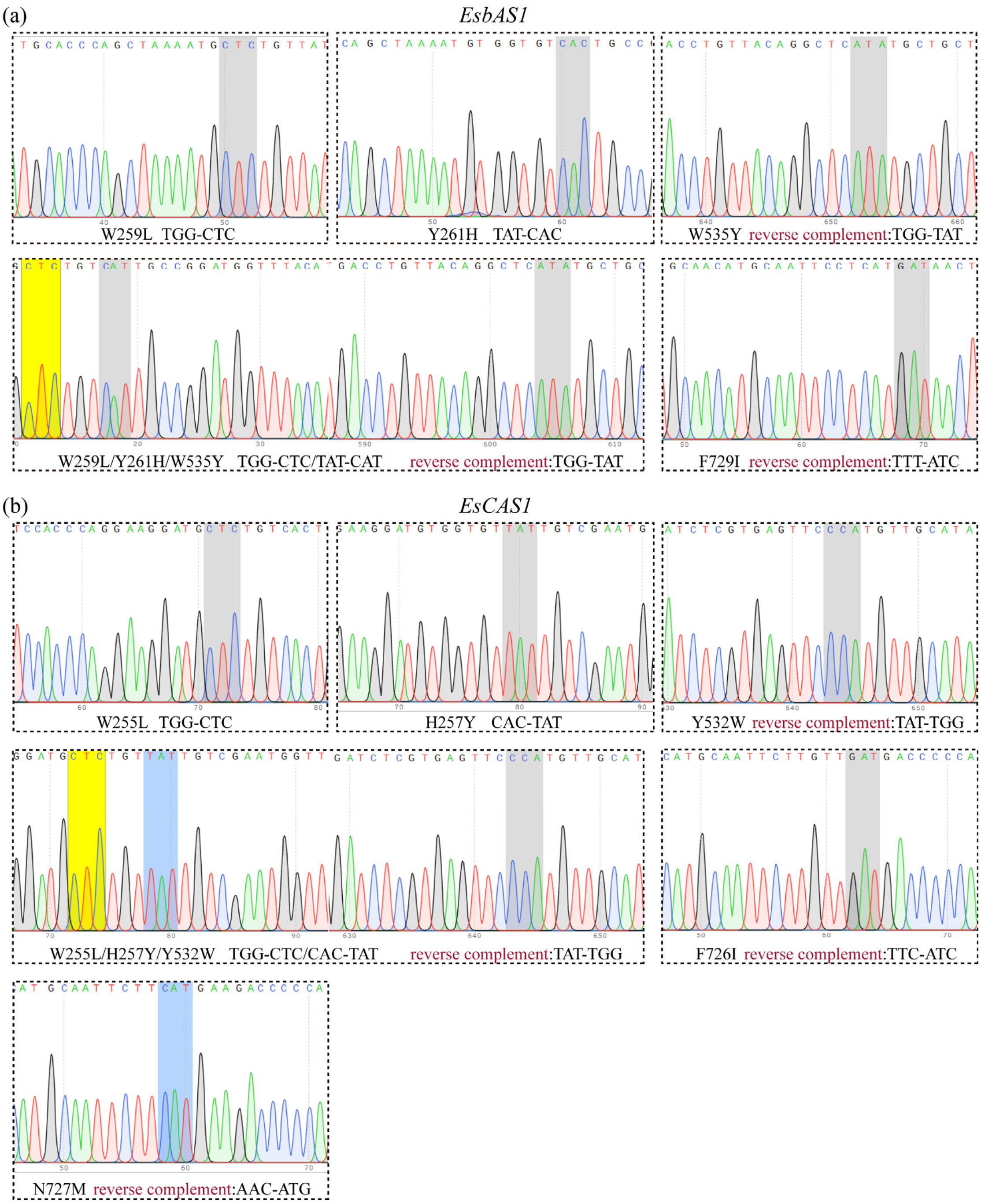

Figure S9 Site-directed mutagenesis sequencing results.(a) Sequencing results of five different mutagenesis combinations of *EsbAS1*.(b) Sequencing results of six different mutagenesis combinations of *EsCAS1*. Reverse complementation represents reverse primer sequencing results.

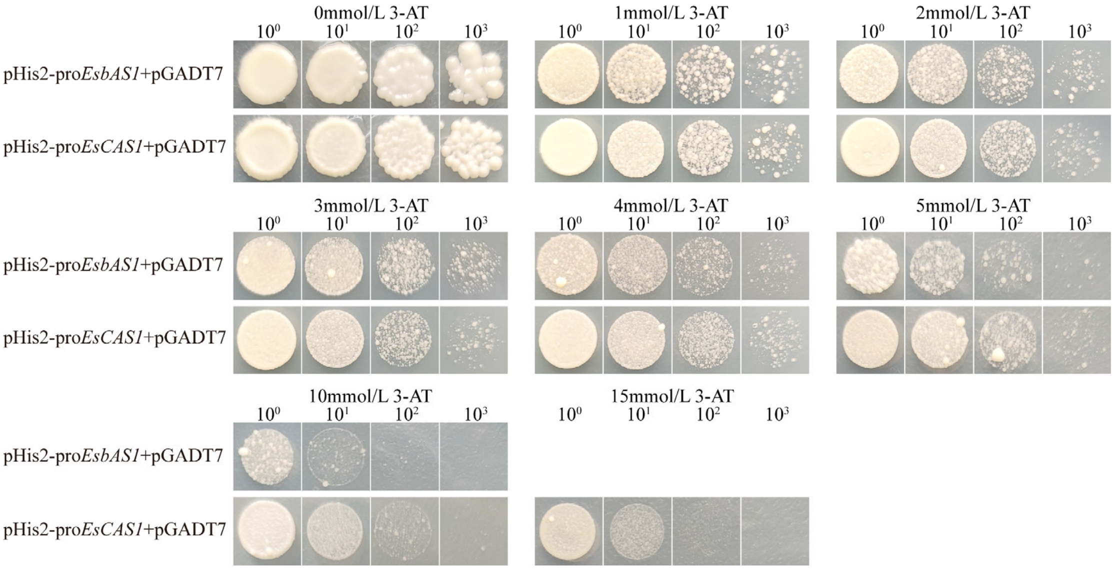

Figure S10 Bait vector self-activation assay. pHis2-pro*EsbAS1*+pGADT7: Y187 yeast cells cotransformed with pHis2-pro*EsbAS1* and pGADT7, pHis2-pro*EsCAS1*+pGADT7: Y187 yeast cells cotransformed with pHis2-pro*EsCAS1* and pGADT7.

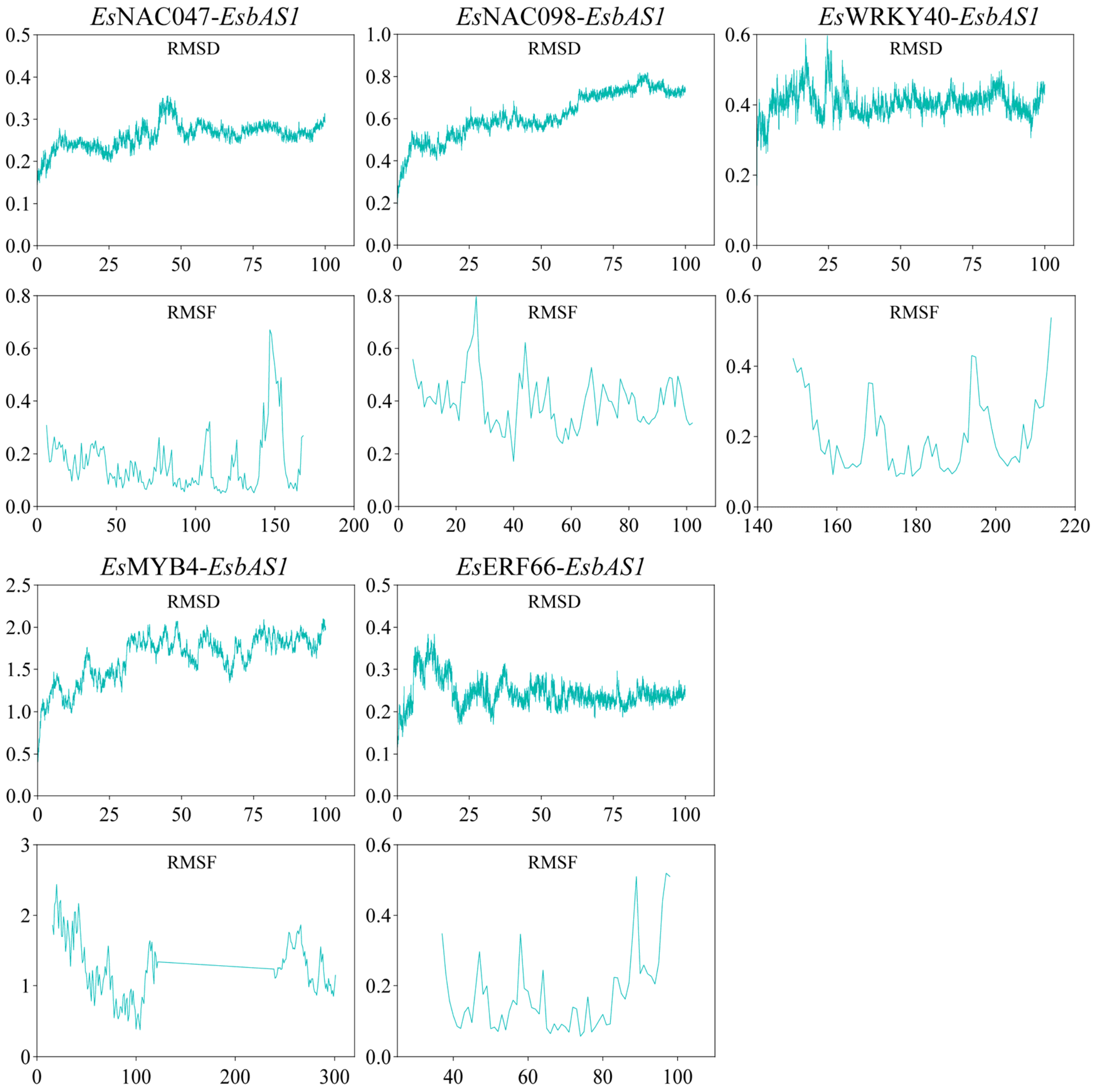

Figure S11 Results of molecular dynamics simulations of *Es*bAS1 with 5 transcription factors.

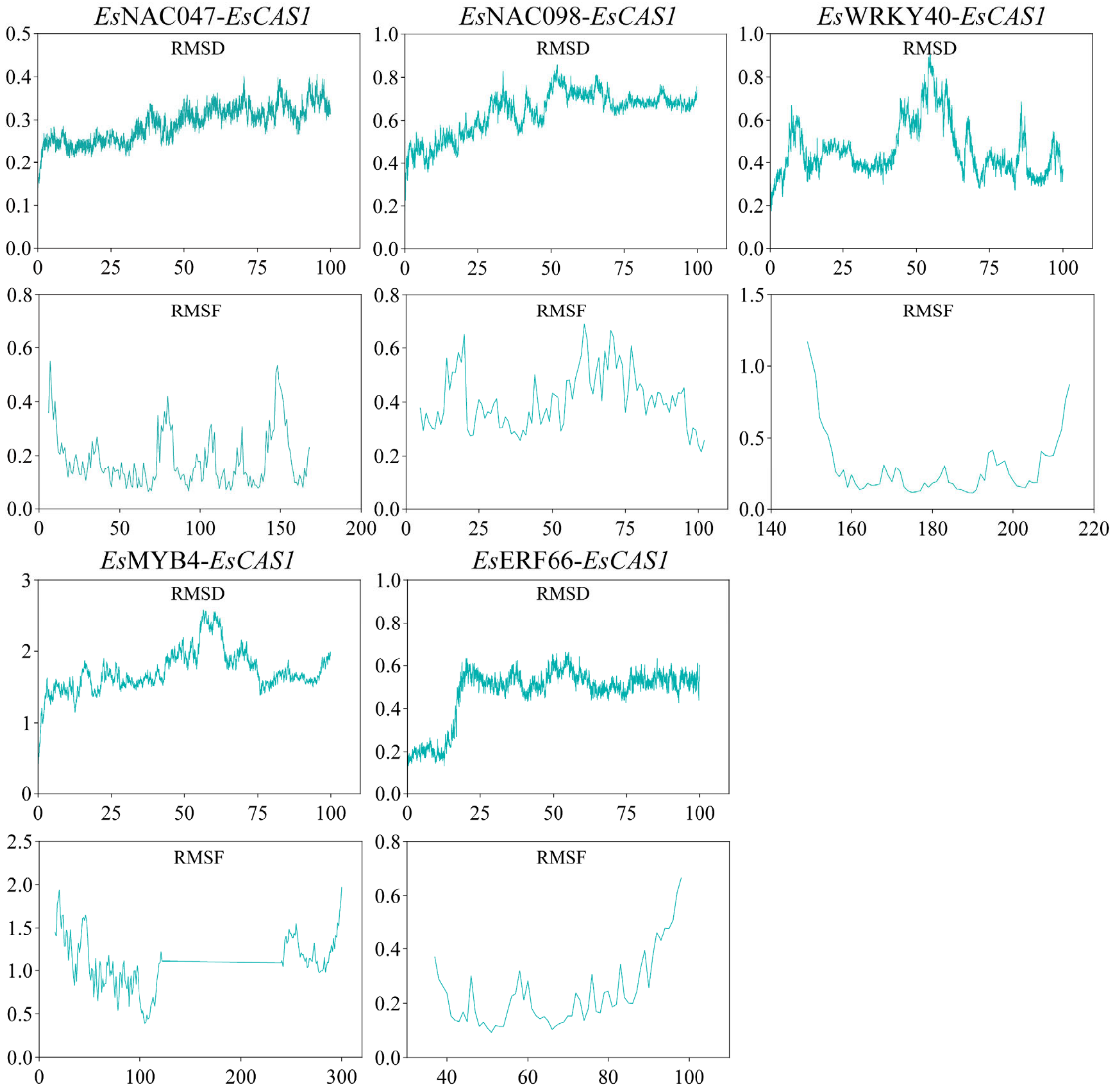

Figure S12 Results of molecular dynamics simulations of *Es*CAS1 with 5 transcription factors.
